# Femtosecond laser-assisted arcuate keratotomy for the management of corneal astigmatism in patients undergoing cataract surgery: Comparison with conventional cataract surgery

**DOI:** 10.3389/fmed.2022.914504

**Published:** 2022-08-25

**Authors:** Hyunmin Ahn, Ikhyun Jun, Kyoung Yul Seo, Eung Kweon Kim, Tae-im Kim

**Affiliations:** ^1^Department of Ophthalmology, Yonsei University College of Medicine, Seoul, South Korea; ^2^Corneal Dystrophy Research Institute, Yonsei University College of Medicine, Seoul, South Korea; ^3^Saevit Eye Hospital, Goyang, South Korea

**Keywords:** arcuate keratotomy, corneal astigmatism, cataract surgery, femtosecond (fs) laser, keratotomy

## Abstract

**Purpose:**

To assess the effects of femtosecond laser arcuate keratotomy with femtosecond laser-assisted cataract surgery in the management of corneal astigmatism, compared with conventional phacoemulsification cataract surgery.

**Design:**

Retrospective comparative interventional case series.

**Methods:**

A total of 2,498 eyes of consecutive patients who presented with 3.00 diopters (D) or under of astigmatism were included. The patients were treated with conventional phacoemulsification cataract surgery (conventional group) and femtosecond laser arcuate keratotomy with femtosecond laser-assisted cataract surgery (femtosecond group).

**Results:**

Surgically induced astigmatism (SIA) was higher in the femtosecond group than the conventional group (0.215, *p* < 0.001). Difference vector (DV) was lower in the femtosecond group (-0.136, *p* < 0.001). The cut-off value of the overcorrection in the femtosecond group was 0.752 D of target induced astigmatism (TIA). For patients with TIA 0.75 D or under, DV and the value of index of success (TIA into DV) were significantly higher in the femtosecond group (*p* = 0.022 and < 0.001). The overcorrection ratios were 48.8% in the conventional and 58.9% in the femtosecond group. (*p* < 0.001). For patients with TIA over 0.75 D, SIA and correction index (TIA into SIA) was higher in femtosecond group (0.310 and 0.250, *p* < 0.001 and < 0.001, respectively). Absolute angle of error was 20.612 ± 18.497 in the femtosecond group and higher than the conventional group (2.778, *p* = 0.010).

**Conclusion:**

Femtosecond laser arcuate keratotomy in cataract surgery was effective in SIA between 0.75 to 3.00 D of corneal astigmatism. However, the overcorrection in the lower astigmatism and angle of error in the higher astigmatism were due to the postoperative corneal astigmatism not decreasing as much as SIA. Overcoming these challenges will lead to better management of corneal astigmatism.

## Introduction

Cataract is the leading cause of blindness worldwide (1), and cataract surgery is among the most common procedures performed in the United States, with more than 30 million patients undergoing surgery each year (2). Phacoemulsification surgery is currently the standard method of treatment for patients with cataracts (1); however, advanced techniques involving the use of multifocal/toric intraocular lenses and other technologies, such as femtosecond laser-assisted cataract surgery (FLACS), have recently become commercially available (3–5).

Corneal astigmatism is a common consideration in cataract surgery, with approximately 40% of patients having astigmatism of more than 1.0 diopter (D) (6). Research has indicated that correction of corneal astigmatism yields better refractive outcomes following cataract surgery (7). Typically, surgical correction of corneal astigmatism involves toric intraocular lens implantation and the creation of corneal arcuate/limbal relaxing incisions, which can be performed either manually or using a femtosecond laser (i.e., FLACS) (4, 8, 9).

Although most previous studies have demonstrated that femtosecond laser-assisted arcuate keratotomy (FL-AK) is effective for correcting corneal astigmatism, there have been several inconsistencies in their results (Supplementary Table 1). Moreover, these studies had insufficient cohorts to conduct a detailed analysis of group differences or determine the factors influencing these differences. As the small sample sizes resulted in a comparative analysis without adjustment for confounders (i.e., independent *t*-test), discrepancies between the results of these previous studies and the effects of corneal astigmatism reduction during FL-AK observed in actual clinical settings are possible.

To overcome these limitations, we performed a detailed and well-controlled analysis using a massive real-world dataset and a statistical method that considered relevant confounders. In this study, we aimed to (a) assess the effectiveness of FL-AK for the management of corneal astigmatism in patients undergoing cataract surgery when compared with conventional phacoemulsification cataract surgery, (b) determine the degrees of preoperative corneal astigmatism for which FL-AK is indicated, and (c) identify the factors related to the effectiveness of FL-AK.

## Materials and methods

### Study design

This retrospective study was performed at Severance Hospital, Yonsei University College of Medicine, between January 2018 and June 2021. The Severance Hospital Clinical Research Ethics Committee approved the protocol of the study (IRB protocol number-4-2021-0525), which was conducted in accordance with the tenets of the Declaration of Helsinki.

### Participants

The study included 2,498 eyes of 1,767 consecutive patients aged ≥ 45 years diagnosed with age-related cataracts and corneal astigmatism ≤ 3.00 D. The surgical method was determined by the patient’s decision-making after the consent of the surgery. Among them, 1,325 eyes of 922 patients were treated with conventional phacoemulsification cataract surgery (conventional group), while 1,173 eyes of 845 patients were treated with FLACS combined with FL-AK (femtosecond group). Patients exhibiting poor compliance during examination and eyes with irregular corneal astigmatism, corneal opacities, previous corneal surgery (including corneal refractive surgery), acute or chronic ophthalmic diseases of the anterior segment, or intraoperative complications were excluded.

### Measurements

All patients underwent a detailed preoperative ophthalmological evaluation, including slit-lamp and fundus examinations. Calculations of intraocular lens power were performed using optical biometry results (IOLMaster 700^®^; Carl Zeiss Meditec AG, Jena, Germany). Corneal measurements and planning of the arcuate keratotomy procedure were based on autokeratometry (Topcon KR-800A; Topcon Corporation, Tokyo, Japan) and Scheimpflug topography (Pentacam^®^; Oculus Inc., Wetzlar, Germany) findings within 2 weeks before surgery. Patients exhibiting a more than 0.3-D difference in mean preoperative corneal astigmatism and those exhibiting axis measurements with differences of 5^°^ or more between autokeratometry and Scheimpflug topography were excluded. Corneal astigmatism was measured *via* autokeratometry 3 months after surgery to assess postoperative outcomes.

### Vector analysis

Vector analysis for corneal astigmatism was conducted using the results of autokeratometry, in accordance with the Alpins method (10). Considering the changes in the astigmatic axis, three vectors were measured: the target-induced astigmatism vector (TIA), defined the astigmatic change that the surgery was intended to induce; the surgically induced astigmatism vector (SIA), defined as the geographic change in corneal astigmatism actually induced by the surgery; and the difference vector (DV), defined as the induced astigmatic change that would enable the initial surgery to achieve its intended target. The magnitude of error (MofE; SIA minus TIA), angle of error (AE, the degree of angle between the TIA and SIA vectors), absolute value of AE (absAE), correction index (CI, SIA divided by TIA), and index of success (IOS; DV divided by TIA) were also measured. The CI > 1 means that the SIA is greater than the TIA, overcorrection. The IOS > 1 means that the corneal astigmatism is increased after surgery, compared to before surgery.

The preoperative corneal astigmatism axis was converted to a range of 0–90 degrees (Axis_90_) and used to classify eyes into three groups: with-the-rule (WTR) astigmatism (0–30 degrees), oblique (OBL) astigmatism (30–60 degrees), and against-the-rule (ATR) astigmatism (60–90 degrees).

### Femtosecond laser system and surgical technique

In the femtosecond group, FLACS and FL-AK were performed using the LenSx^®^ femtosecond system (Alcon laboratory, Inc., Texas, United States). Intra-operative alignment of the corneal astigmatism axis was performed using a corneal topography system (Verion^®^ image guide system; Alcon laboratory, Inc.; Texas, United States) immediately before surgery. A single arcuate keratotomy incision was paired in the opposite meridian. The depth of the astigmatic keratotomy was set at 80% corneal thickness according to a modified Donnenfeld limbal relaxing nomogram (11–14). The diameter of the optical zone was set to 8.0 mm. For FLACS, the laser was also used to perform a 5.0–5.3-mm capsulotomy and lens fragmentation. The femtosecond system was not used to make the main phacoemulsification incision or the peripheral incision.

In both groups, the clear corneal incision at the temporal side was created using a 2.80-mm keratome, and the anterior capsule button was removed. In the conventional group, capsulotomy was performed using forceps or a capsulotomy needle. Phacoemulsification was performed under local anesthesia in both groups using an Infiniti^®^ system (Alcon laboratory, Inc.; Texas, United States). All operations were performed by an experienced surgeon (T.I.K). Patients were instructed to perform 1 month of postoperative care, which included instillation of eye drops, in accordance with the standard protocol for cataract surgery. Patients who did not follow the surgical instructions were excluded.

### Statistical analysis

The data were analyzed using descriptive statistics, and the mean values and standard deviations were computed for each variable. Differences between the groups were initially assessed using independent *t*-tests, which were adjusted for age, sex, laterality, preoperative corneal refractive power (Km), preoperative corneal astigmatism axis, and TIA. Linear regression analyses were used examine the association between TIA and SIA in each group. Using the cut-off value for overcorrection (i.e., SIA > TIA) identified in the linear regression analysis, stratified analyses were conducted for patients in the femtosecond group. Lower and higher TIA were defined as TIA values under and over the cut-off value, respectively. *P*-values < 0.05 were considered statistically significant.

## Results

The two groups exhibited no significant differences in baseline demographic or ophthalmological characteristics, including age, sex, laterality distribution, preoperative corneal refractive power (Km), preoperative corneal astigmatism, and axis (Table 1).

**TABLE 1:** Characteristics of patients in the conventional and femtosecond groups.

	Conventional group (*n* = 1,325)	Femtosecond group (*n* = 1,173)	*p*
Age	66.47 ± 10.94	66.71 ± 12.78	0.623
Sex (female)	824 (62.2%)	769 (65.6%)	0.087
Laterality (right, %)	674 (50.9%)	599 (51.1%)	0.936
Preoperative corneal refractive power (Km, D)	44.14 ± 1.88	44.20 ± 1.75	0.328
Preoperative corneal astigmatism	0.85 ± 0.58	0.85 ± 0.58	0.983
Preoperative corneal astigmatism axis (Axis_90_)	43.45 ± 31.78	45.49 ± 31.90	0.085
Preoperative corneal astigmatism group			0.404
Against-the-rule	580 (43.8%)	487 (41.5%)	–
Oblique	228 (17.2%)	198 (16.9%)	–
With-the-rule	517 (39.0%)	488 (41.6%)	–

TIA was 0.845 ± 0.579 D in the conventional group and 0.846 ± 0.578 D in the femtosecond group (*p* = 0.983). However, SIA was significantly higher in the femtosecond group than in the conventional group (adjusted difference = 0.215, *p* < 0.001), whereas DV was significantly lower in the femtosecond group (adjusted difference = -0.136, *p* < 0.001). IOS did not significantly differ between the groups (adjusted difference = -0.047, *p* = 0.668) (Table 2A).

**TABLE 2:** Vector analysis of postoperative corneal astigmatism.

A. Overall.									
	Conventional (*n* = 1,325)		Femtosecond (*n* = 1,173)				Adjusted^†^	Adjusted^†^
	Mean	*SD*	Mean	*SD*	Diff	*p*	Diff	*p*
TIA	0.845	0.579	0.846	0.578	0.001	0.983	-	-
SIA	0.631	0.494	0.886	0.819	0.254	<0.001*	0.215	<0.001*
DV	0.913	0.823	0.803	0.772	-0.110	<0.001*	-0.136	<0.001*
AE	0.434	27.771	1.063	30.158	0.628	0.596	0.192	0.903
absAE	21.041	18.120	22.673	19.903	1.632	0.036*	1.634	0.033*
MofE	-0.135	0.777	0.041	0.862	0.175	<0.001*	0.215	<0.001*
CI	0.748	0.823	1.037	1.075	0.289	<0.001*	0.319	<0.001*
IOS	1.082	1.052	0.951	1.012	-0.131	0.002*	-0.047	0.668

**B. Preoperative astigmatism ≤ 0.75 D.**								
	**Conventional (*n* = 813)**		**Femtosecond (*n* = 693)**				**Adjusted** ^†^	**Adjusted** ^†^
	**Mean**	***SD***	**Mean**	***SD***	**Diff**	***p***	**Diff**	***p***

TIA	0.486	0.214	0.485	0.219	–0.001	0.998	–	–
SIA	0.607	0.658	0.728	0.688	0.121	0.001*	0.234	<0.001*
DV	0.700	0.676	0.713	0.638	0.012	0.719	0.099	0.022*
AE	1.021	30.733	0.229	31.790	–0.792	0.623	–1.209	0.311
absAE	24.356	18.961	24.098	20.714	–0.258	0.378	–0.864	0.534
MofE	0.122	0.684	0.266	0.702	0.145	<0.001*	0.234	<0.001*
CI	1.418	1.685	1.776	2.018	0.358	<0.001*	0.316	<0.001*
IOS	1.598	1.685	1.744	1.965	0.146	0.132	0.249	<0.001*

**C. Preoperative astigmatism > 0.75 D.**								
	**Conventional (*n* = 512)**		**Femtosecond (*n* = 480)**				**Adjusted** ^†^	**Adjusted** ^†^
	**Mean**	***SD***	**Mean**	***SD***	**Diff**	***p***	**Diff**	***p***

TIA	1.395	0.420	1.400	0.461	–0.004	0.879	–	–
SIA	0.790	0.591	1.114	0.932	0.324	<0.001*	0.310	<0.001*
DV	1.170	0.631	0.933	0.842	–0.237	<0.001*	-0.203	<0.001*
AE	0.242	24.321	2.268	27.618	2.026	0.133	0.954	0.670
absAE	18.224	16.087	20.612	18.497	2.388	0.016*	2.778	0.010*
MofE	–0.608	0.993	–0.286	0.963	–0.322	<0.001*	–0.310	<0.001*
CI	0.596	0.462	0.840	0.761	0.244	0.001*	0.250	<0.001*
IOS	0.848	0.442	0.668	0.599	–0.182	<0.001*	–0.186	<0.001*

*Statistically significant.

^†^Adjusted for age, sex, laterality, surgeon, Km, preoperative corneal astigmatism axis, and TIA.

absAE, absolute angle of error; AE, angle of error; CI, correction index; DV, difference vector; IOS, index of success; MofE, magnitude of error; SIA, surgically induced astigmatism; TIA, target induced astigmatism.

The cut-off TIA value for overcorrection in the femtosecond group was 0.752 (95% confidence interval: 0.512–0.992). The linear regression Equation between SIA and TIA in the femtosecond group was as follows (Supplementary Image 1):

*SIA* = 0.457+0.392*TIA* (R = 0.272, *p* < 0.001)

SIA and CI values were higher in the femtosecond group than in the conventional group among patients with both lower and higher TIA (all *p* < 0.001). In patients with lower TIA, the mean DV value was higher in the femtosecond group than in the conventional group (adjusted difference = 0.099, *p* = 0.022). The MofE values were 0.122 ± 0.684 D and 0.266 ± 0.702 D in the conventional and femtosecond groups, respectively (adjusted difference = 0.145, *p* < 0.001) (Table 2B). In patients with higher TIA, the DV value was significantly lower in the femtosecond group than in the conventional group (adjusted difference = -0.203, *p* < 0.001). The femtosecond group also had higher absolute AE values than the conventional group (adjusted difference = 2.778, *p* = 0.010) and an IOS of 0.668 ± 0.599 (Table 2C).

For both patients with lower and higher TIA, the overcorrection ratio was significantly higher in the femtosecond group than in the conventional group (*p* < 0.001 and < 0.001, respectively) (Table 3A). However, the overcorrection ratio did not significantly differ among the preoperative corneal astigmatism axis subgroups (ATR vs. OBL vs. WTR) when the analysis was restricted to patients of the femtosecond group with lower TIA (*p* = 0.643) (Table 3B).

**TABLE 3:** Overcorrection ratio in stratified analyses for target induced astigmatism ≤ 0.75 diopters.

A. Treatment groups.						
		**Conventional**		**Femtosecond**		**Total**
Overcorrection		397 (48.8%)		408 (58.9%)		805 (53.5%)
No overcorrection		416 (51.2%)		285 (41.1%)		701 (46.5%)
Total		813 (54.0%)		693 (46.0%)		1,506 (100%)
						*p* < 0.001*

**B. Femtosecond subgroups based on preoperative corneal astigmatism axis.**						
	**With-the-rule**		**Oblique**	**Against-the-rule**		**Total**
Overcorrection	98 (40.2%)		58 (38.4%)	108 (36.2%)		285 (41.4%)
No overcorrection	146 (59.8%)		93 (61.6%)	190 (63.8%)		408 (58.9%)
Total	244 (35.2%)		151 (21.8%)	298 (43.0%)		693 (100%)
						*p* = 0.643

*Statistically significant.

A linear regression analysis adjusted for TIA and SIA indicated that absolute AE was significantly associated with DV among patients in the femtosecond group with higher TIA [B = 0.014 (95% confidence interval: 0.011–0.018), *p* < 0.001] (Table 4).

**TABLE 4:** Linear regression analysis of the difference vector in patients of the femtosecond group with target induced astigmatism over 0.75 D (adjusted *R*^2^ = 0.406, *p* < 0.001*).

			95% Confidence Interval			
	B	ß	Lower	Upper	*P*	VIF
(Constant)	–0.054	–	–0.263	0.154	0.609	–
absAE	0.014	0.315	0.011	0.018	<0.001*	1.011
SIA	0.461	0.510	0.397	0.525	<0.001*	1.035
TIA	0.340	0.186	0.210	0.470	<0.001*	1.046

*Statistically significant.

absAE, absolute angle of error; SIA, surgically induced astigmatism; TIA, target induced astigmatism.

Overall, 72.0% of the patients in the femtosecond group exhibited decreased corneal astigmatism postoperatively. When compared with the preoperative values, 8.4% exhibited a decrease of more than 75%, while 22.9% exhibited a decrease of more than 50% (Supplementary Image 2). This Venn diagram shows the distribution of DV by TIA, SIA, and AE. Depending on AE, even with sufficient SIA compared to TIA, DV cannot reach TIA, and can even be greater than TIA.

## Discussion

In this study, we investigated the effect of FL-AK in managing corneal astigmatism during cataract surgery using real-world data for 2,498 eyes. In the overall cohort, our findings indicate that the femtosecond group had higher SIA but lower postoperative corneal astigmatism than the conventional group. However, the MofE (TIA subtracted from SIA) was positive, and the CI value (SIA divided by TIA) was over 1, indicating that overcorrection was common in the femtosecond group. The difference in SIA between the conventional and femtosecond groups was 0.254, but the absolute difference in postoperative corneal astigmatism (0.110. SIA) was higher in the femtosecond group; however, this effect was not fully reflected in the degree of postoperative corneal astigmatism. Linear regression analysis between TIA and SIA also indicated that the TIA cut-off value for overcorrection was 0.752 D, which is within the range reported in previous studies (15, 16).

In patients with TIA values of 0.75 D or under, FL-AK induced significant overcorrection and reductions in corneal astigmatism when compared with the conventional method. In a previous study that also included patients with relatively lower preoperative corneal astigmatism, overcorrection was related to the preoperative corneal astigmatism axis (17), as observed in the current study. However, the overcorrection rates did not significantly differ between the groups (*p* = 0.643). In another study, the authors reported that, in patients with a relatively lower degree of corneal astigmatism, FL-AK outcomes were influenced by preoperative corneal astigmatism and uncorrected visual acuity (18). The authors of that study utilized a novel formula to reduce the corneal incision arc by 20–30%, and the novel formula was more effective in correcting low astigmatism than the pre-existing method. Thus, reducing the corneal incision arc indicated by the existing formula may aid in lowering preoperative astigmatism in these patients.

**FIGURE 1:**
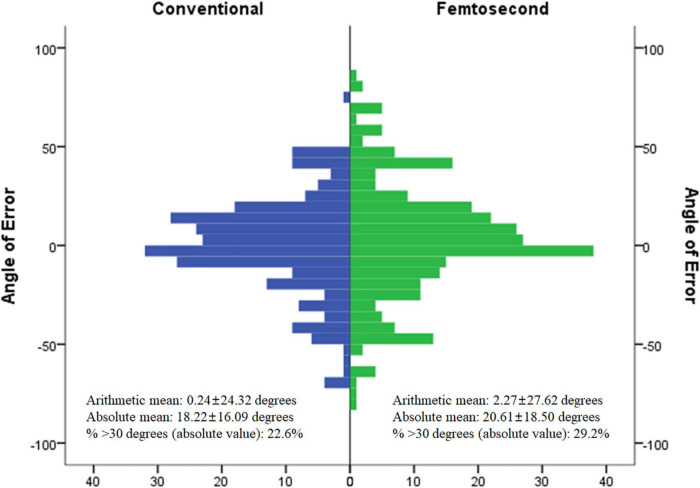
Angle of error between conventional group and femtosecond group in target induced astigmatism over 0.75 D.

In patients with TIA values > 0.75 D, the goal indicated by the FL-AK nomogram was 70–80% correction of preoperative corneal astigmatism, and SIA values indicated that 84% correction had been achieved. However, approximately 67% of preoperative corneal astigmatism remained in the FL-AK group, similar to findings reported in previous studies (0.47–0.71) (11, 15, 19, 20). IOS reflects the amount postoperative corneal astigmatism remaining when compared with the preoperative state. We focused on IOS because we assumed it to underlie the central conflict between the effects of FL-AK reported in previous studies (which focused on SIA) and the actual clinical situation. In the current study, postoperative corneal astigmatism was not defined based on the arithmetic difference between preoperative corneal astigmatism and SIA (21). Our findings suggest that AE (i.e., the angle between TIA and SIA) was the primary cause of these discrepancies (Figure 1). The absAE in the femtosecond group was 20.6, which is within the range of 17.5–25.1 reported in previous studies (9, 15). Considering the relationships among the vectors (TIA, SIA, and DV), the evidence indicates that AE is among the major factors influencing postoperative corneal astigmatism in patients treated with FL-AK during cataract surgery (10, 21).

In previous studies, torque was regarded as an ineffective component of the SIA vector (21–23), and the direction of SIA was tilted to the induced direction. Moreover, several studies have highlighted the importance of the reference axis in arcuate keratotomy (24, 25). Further studies are required to determine the precise factors affecting AE and to develop a novel nomogram incorporating AE, as this will help to improve the effectiveness of FL-AK in patients undergoing FLACS.

In conclusion, our findings indicate that cataract surgery with FL-AK resulted in significantly increased SIA but that it was effective in correcting preoperative corneal astigmatism > 0.75 D when compared with conventional phacoemulsification cataract surgery. However, overcorrection in patients with a lower degree of astigmatism and the angle of error in patients with higher astigmatism may have inhibited improvements in postoperative corneal astigmatism. Future studies should aim to overcome these challenges to achieve better efficacy in managing corneal astigmatism.

## Data availability statement

The raw data supporting the conclusions of this article will be made available by the authors, without undue reservation.

## Ethics statement

The studies involving human participants were reviewed and approved by the Severance Hospital Clinical Research Ethics Committee. Written informed consent for participation was not required for this study in accordance with the national legislation and the institutional requirements.

## Author contributions

T-IK conceived and designed the analysis. HA collected the data, performed the analysis, and wrote the manuscript. IJ, KS, EK, and T-IK contributed to the data. All authors contributed to the article and approved the submitted version.

## Abbreviations

absAE: absolute value of angle of error
AE: angle of error
ATR: against-the-rule astigmatism
CI: correction index
D: diopters
DV: difference vector
FLACS: femtosecond laser-assisted cataract surgery
FL-AK: femtosecond laser-assisted arcuate keratotomy
IOS: index of success
MofE: magnitude of error
OBL: oblique astigmatism
SIA: surgically induced astigmatism
TIA: target induced astigmatism
WTR: with-the-rule astigmatism.
