# Supplementary figures and images for: Femtosecond laser-assisted arcuate keratotomy for the management of corneal astigmatism in patients undergoing cataract surgery: Comparison with conventional cataract surgery
Source: Front Med (Lausanne). 2022 Aug 25;9:914504. doi: 10.3389/fmed.2022.914504 (PMC9453263; doi:10.3389/fmed.2022.914504)

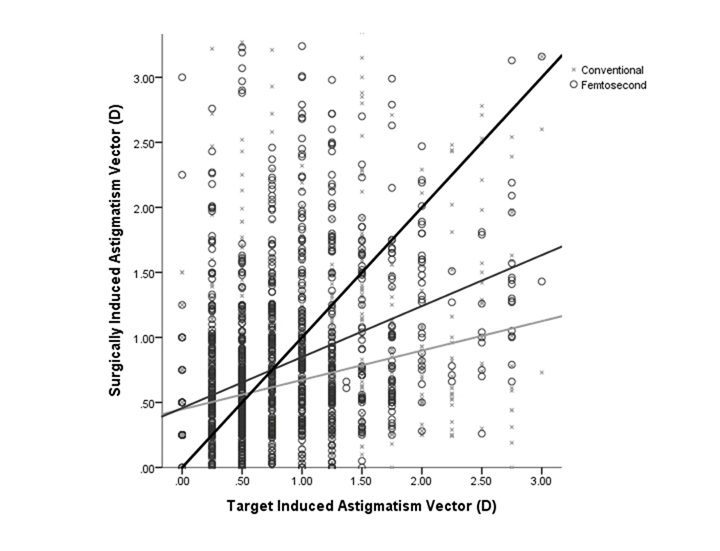

Supplement: Supplementary file 1 [file Image_1.tif]

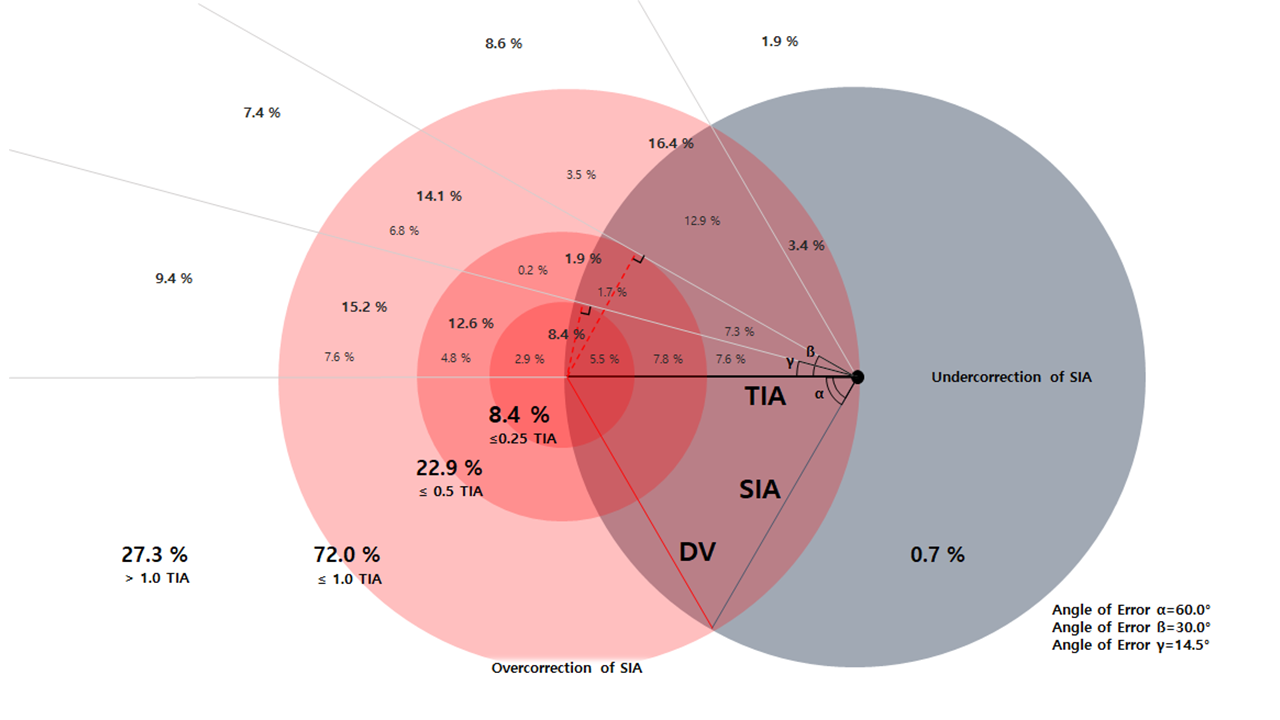

Supplement: Supplementary file 2 [file Image_2.tif]

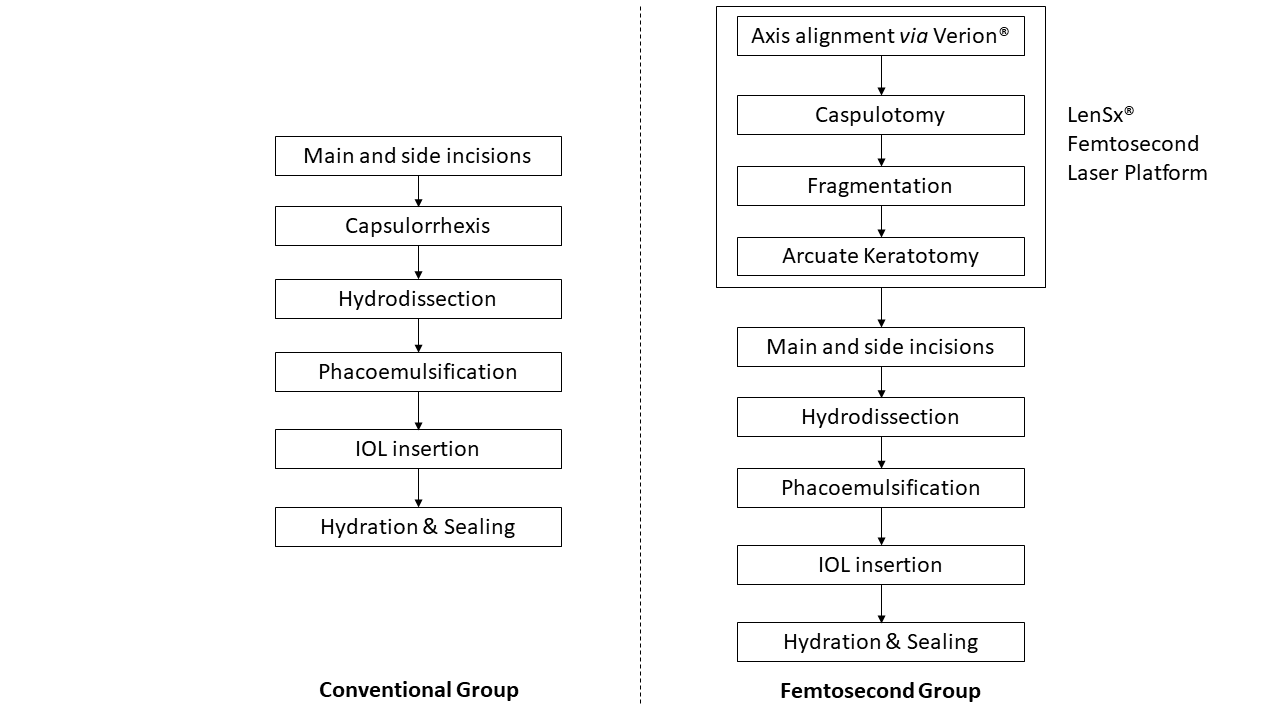

Supplement: Supplementary file 3 [file Image_3.TIF]
