# Supplementary material for: Femtosecond laser-assisted arcuate keratotomy for the management of corneal astigmatism in patients undergoing cataract surgery: Comparison with conventional cataract surgery
Source: Front Med (Lausanne). 2022 Aug 25;9:914504. doi: 10.3389/fmed.2022.914504 (PMC9453263; doi:10.3389/fmed.2022.914504)
Supplement: Supplementary file 4 [file Table_1.DOCX]

Supplementary Material

**Femtosecond Laser-assisted Arcuate Keratotomy for the Management of Corneal Astigmatism in Patients Undergoing Cataract Surgery: Comparison with Conventional Cataract Surgery**

**Supplementary Table 1.** Previous articles related to femtosecond laser-assisted arcuate keratotomy

| Authors | Year | Design | Number of eyes  (Number of patients) | AST  Range (D) | | | Laser System | Nomogram | Arc  Depth | Arc Diameter | AK  Type | FU |
| --- | --- | --- | --- | --- | --- | --- | --- | --- | --- | --- | --- | --- |
| Chan TCY, et al.[15] | 2015 | Retrospective | 53 (53) | (0.50) | - | (2.75) | Victus® | modified Wallace | 450 μm | 8.0 mm | Penetrating | 2M |
| Yoo A, et al.[26] | 2015 | Retrospective | 48 (48) | 1.00 | - | 3.00 | IntraLase® | modified Donnenfeld | 85% CT | 9.0 mm | Penetrating | 5M |
| Chan TCY, et al.[19] | 2016 | Retrospective | 50 (50) | (0.50) | - | 2.50 | Victus® | modified Wallace | 450 μm | 8.0 mm | Penetrating | 2Y |
| Day AC, et al.[27] | 2016 | Prospective | 196 (133) | 0.70 | - |  | Catalys® | Personal^†^ | 20-80% CT | 8.0 mm | IS | 1M |
| Baharozian CJ, et al.[17] | 2017 | Retrospective | 161 (116) | 0.25 |  | 2.00 | Catalys® | modified Donnenfeld | 80% CT | 9.0 mm | Penetrating | 1-2M |
| Roberts HW, et al.[8] | 2018 | RCT | 51 (51) | 0.90 | - |  | LenSx® | Personal^†^ | 20-80% CT | 8.0 mm | IS | 1M |
| Visco DM, et al.[16] | 2019 | Retrospective | 189 (143) | 0.50 | - | 2.00 | Lensar® | Nichamin-Woodcock | 90% CT | 8.6 mm | Penetrating | 3M |
| Ganesh S, et al.[11] | 2019 | RCT | 50 (50) | 0.75 | - | 2.00 | Catalys® | modified Donnenfeld  Personal^†^ | 80% CT  20-80% CT | 8.0 mm | Penetrating, IS | 6M |
| Hiep NX, et al.[12] | 2019 | Prospective | 45 (33) | 0.50 | - |  | LenSx® | modified Donnenfeld | 85% CT | 8.0 mm | Penetrating | 3M |
| Lee JA, et al.[28] | 2019 | Retrospective | 49 (39) | 0.75 | - |  | Catalys® | Personal^†^ | 20-80% CT | 8.0 mm | IS | 1M |
| Chan TCY, et al.[20] | 2020 | Retrospective | 44 (44) | 0.50 | - | 2.50 | Victus® | modified Wallace | 450 μm | 8.0 mm | Penetrating | 5Y |
| Lopes D, et al.[13] | 2020 | Retrospective | 40 (20) | 0.70 | - | 2.00 | Catalys® | modified Donnenfeld  Personal^†^ | 80% CT  20-80% CT | 9.0 mm  8.0 mm | Penetrating,  IS | 5M |
| Rani K, et al.[29] | 2020 | Prospective | 80 (70) | 0.40 | - | 1.50 | Catalys® | modified Donnenfeld | 80% CT | 8.0 mm | Penetrating | 3M |
| Wortz G, et al.[18] | 2020 | Retrospective | 224 (224) |  | - | 1.00 | Catalys® | Wörtz-Gupta™ formula  modified Donnenfeld | 80% CT | 9.0 mm | Penetrating | 4W |
| Schwarzenbacher L, et al.[9] | 2021 | Prospective | 43 (43) | 1.00 | - | 3.00 | FEMTO LDV Z8 FSL® | Castrop Femto AK^‡^ | 80% CT | 8.5 mm | Penetrating | 1Y |

† Stevens J. Intrastromal AK nomogram calculator v3 2015. Available at: www.femtoemulsification.com.

‡ Hoffmann P, Lindemann C, Abraham M. [Development of a nomogram for fs-Laser arcuate Incisions]. In: Deutschsprachigen Gesellschaft für Intraokularlinsen-Implantation, Interventionelle und Refraktive Chirurgie; 2014.

AK, arcuate keratotomy; AST, corneal astigmatism; CT, corneal thickness; D, diopter; FU, follow-up; IS, intrastromal; RCT, randomized clinical trial.

**Supplementary Table 1 (continued).** Previous articles related to femtosecond laser-assisted arcuate keratotomy

| Authors | TIA | SIA | DV | AE | aAE | ME | CI | IOS | SIA/TIA † | | Cut-off‡ | Postop ≤0.5 D | Overcorrection |
| --- | --- | --- | --- | --- | --- | --- | --- | --- | --- | --- | --- | --- | --- |
| Chan TCY, et al.[15] | 1.33 | 1.20 | 0.87 | 0.1 | 17.5 | -0.13 | 0.86 | 0.62 | Y = 0.4960X + 0.5384 | R^2^=0.17 | (1.068) | (42.6%) | (50.0%) |
| Yoo A, et al.[26] | 1.30 | (1.27) | 0.87 | -3.7 | - | -0.03 | 1.14 | - | - | - | - | - | - |
| Chan TCY, et al.[19] | 1.35 | 1.23 | 0.74 | - | 14.0 | -0.12 | 0.85 | 0.51 | - | - | - | (24.0%) | (30.0%) |
| Day AC, et al.[27] | 1.21 | 0.74 | 0.74 | 3.0 | - | -0.47 | 0.63 | 0.63 | Y = 0.4280X + 0.2183 | R^2^=0.20 | (0.382) | 32.1% | 10.7% |
| Baharozian CJ, et al.[17] | 0.86 | - | 0.63 | - | - | - | (0.69) | - | - | - | - | 49.7% | (21.7%) |
| Roberts HW, et al.[8] | 1.38 | 1.23 | 0.89 | 2.4 | - | (-0.15) | 0.73 | 0.65 | Y = 0.7698X + 0.1628 | R^2^=0.16 | (0.707) | (58.8%) | (29.4%) |
| Visco DM, et al.[16] | 0.92 | - | 0.14 | - | - | - | - | - | Y = 0.9362X + 0.0163 | R^2^=0.79 | (0.255) | 95.8% | - |
| Ganesh S, et al.[11] (Penetrate) | 1.16 | 1.23 | 0.69 | - | 16.5 | 0.07 | 0.95 | 0.71 | - | - | - | - | 48.0% |
| Ganesh S, et al.[11] (Intrastromal) | 1.50 | 1.08 | 0.93 | - | 13.2 | -0.42 | 0.55 | 0.59 | - | - | - | - | 28.0% |
| Hiep NX, et al.[12] | 1.65 | 1.05 | 0.59 | - | - | - | 0.64 | - | - | - | - | - | (0.0%) |
| Lee JA, et al.[28] | 1.10 | - | 0.59 | - | - | - | - | - | - | - | - | - | - |
| Chan TCY, et al.[20] | 1.40 | 1.28 | 0.70 | 1.7 | 13.4 | -0.12 | 0.85 | 0.47 | - | - | - | (70.5%) | (43.1%) |
| Lopes D, et al.[13] (Penetrate) | 1.25 | 1.35 | 0.83 | -12.0 | - | - | 0.83 | - | - | - | - | 30.0% | 25.0% |
| Lopes D, et al.[13] (Intrastromal) | 1.26 | 0.76 | 0.75 | 5.5 | - | - | 0.68 | - | - | - | - | 40.0% | 5.0% |
| Rani K, et al.[29] | (0.85) | - | (0.50) | - | - | - | - | - | - | - | - | 68.8% | - |
| Wortz G, et al.[18] | 0.61 | - | 0.27 | - | - | - | - | - | - | - | - | 88.7% | - |
| Schwarxenbacher L et al.[9] | 1.24 | 0.95 | 0.68 | - | 25.1 | -0.28 | - | - | - | - | - | - | - |
| Penetrate* (n=858) | 1.32  [1.02] | 1.18 | 0.74  [0.49] | -1.8 | 17.2 | -0.18 | 0.80 | 0.56 | - | - | 0.433 | 61.0%  [70.7%] | 33.9% |
| Intrastromal* (n=306) | 1.08 | 0.86 | 0.75 | 3.1 | 13.2 | -0.26 | 0.64 | 0.63 | - | - | 0.449 | 37.7% | 15.2% |

( ) Estimated value based on the values listed in the article. [ ] Study including patients with lower astigmatism, Wortz G, et al. and Baharozian CJ, et al. * Calculation with adjustment for arithmetic mean, standard deviation, and the number of cases.

† Linear regression equation between TIA and SIA. ‡ Cut-off value for overcorrection calculated based on the linear regression equation between TIA and SIA

aAE, absolute angle of error; AE, angle of error; Coefficient CI, confidence interval; CI, correction index; DV, difference vector; IOS, index of success; ME, magnitude of error; SIA, surgically induced astigmatism; TIA, target induced astigmatism.

**Supplementary Image 1.** Flow diagram of the surgical processes in the conventional and the femtosecond groups.

**Supplementary Image 2.** Scatterplot of target induced astigmatism (TIA) versus surgically induced astigmatism (SIA) in the conventional (*c*) and femtosecond groups (*f*). The thick black line represents the point at which TIA equals SIA*.* Equation for the conventional group: $SIAc=0.447+0.226\times TIAc$ (R=0.245, p<0.001). Equation for the femtosecond group: $SIAf=0.457+0.392\times TIAf$ (R=0.272, p<0.001)

**Supplementary Image 3.** Difference vectors in the femtosecond group
